# The global challenge of glaucoma

**Published:** 2022-01-31

**Authors:** Victor H Hu

**Affiliations:** 1 Assistant Clinical Professor: International Centre for Eye Health, London School of Hygiene & Tropical Medicine and Consultant Ophthalmologist, Mid Cheshire NHS Hospitals, UK.

**The *Lancet Global Health* Commission on Global Eye Health, a wide-ranging report synthesising new and existing research across many aspects of eye health, looked at the global challenges of offering glaucoma care to everyone who needs it.**

**Figure:**
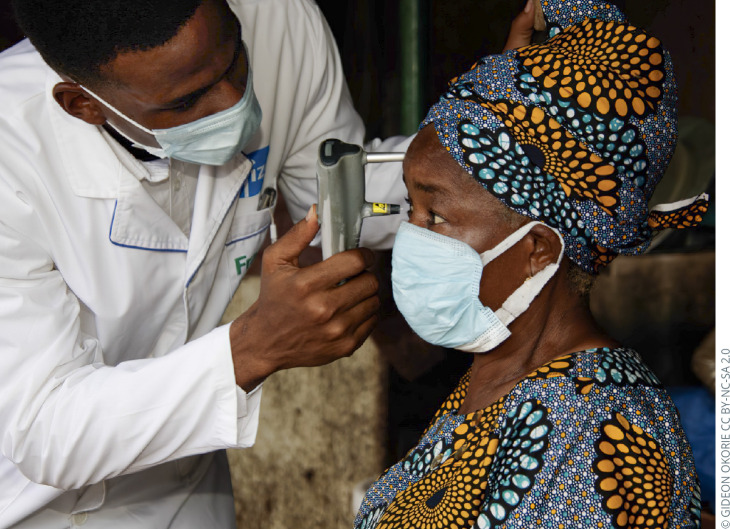
Testing intraocular pressure during community screening. **NIGERIA**

Glaucoma is a major cause of irreversible blindness worldwide. It also results in substantial disability, even before people become blind from it, but remains under-treated globally. In most surveys carried out in high-income countries, over 50% of people found to have glaucoma had not been diagnosed and are therefore not receiving treatment. In low-income and/or middle-income countries (LMICs), this rises to over 90%. This high percentage is because glaucoma is mostly asymptomatic until relatively late in the disease. In LMICs, as many as 35% of people diagnosed with glaucoma are already blind as a result – it is too late for them to benefit from effective interventions that would have prevented vision loss.

Whereas cataract has a one-stop solution (cataract surgery), glaucoma requires more complex management strategies because of its chronic nature and complexity. In the absence of simple and affordable diagnostic and treatment solutions, the global eye health community has not prioritised glaucoma; for example, when VISION 2020 was being developed more than 20 years ago. There are several crucial issues.

First, there is a need to provide effective treatments that prevent glaucoma progression and, maybe someday, restore visual function to those with glaucomatous damage. Lowering intraocular pressure (IOP) slows, and in some cases stops, glaucoma progression, but doing so safely and effectively remains a challenge. The current treatment is often long-term topical eye drops, but poor compliance and ongoing costs are major challenges in low-resource settings. Laser trabeculoplasty, which can be administered in a single session, is an effective strategy that has shown effectiveness in such settings. Unfortunately, it rarely provides lifetime control of IOP. Although there is hope that in the future more effective surgical or laser approaches will provide safe and sustained pressure lowering, more work needs to be done.

Second, individuals need to be monitored to determine whether their glaucoma is progressing so that treatment can be adjusted as needed. Monitoring presents challenges for more remote and resource-limited populations, but home-based monitoring using off-the-shelf technology might become available in the near future. The growth of vision centres in India and elsewhere, staffed by mid-level ophthalmic personnel and supported remotely by ophthalmologists, is an example of how to provide ongoing monitoring and care for people living in remote settings.

**“Individuals need to be monitored to determine whether their glaucoma is progressing so that treatment can be adjusted as needed.”**

Third, affordable and effective screening approaches are needed to enable identification of individuals at risk of sight loss. Major advances in the automated grading of optic disc photographs have led to highly accurate glaucoma diagnoses on the basis of a single photo. Widespread use of screening using fundus imaging, with artificial intelligence-assisted grading, could allow glaucoma to be diagnosed alongside the other major causes of blindness at low cost. Implementation studies are needed to determine how and where to apply these new tools.

Innovation in glaucoma detection and management could catalyse a new care model in which earlier detection and effective long-term IOP lowering, combined with remote monitoring, can prevent unnecessary blindness worldwide. To reach this goal, the global eye care community must include glaucoma in eye care planning, recognising that the patient is a central partner in its management. Many important research questions remain unresolved and require substantial investment and a concerted global effort to answer.

Adapted for the *Community Eye Health Journal* from Panel 5, Lancet Global Health, *Commission on Global Eye Health*, p. 519.

Where to access the report: **https://globaleyehealth commission.org**

